# A noteworthy case of rewilding Chinese yew from a garden population in eastern China

**DOI:** 10.7717/peerj.12341

**Published:** 2021-10-19

**Authors:** Kaidi Li, Guangfu Zhang, Ying Zhang, M. Patrick Griffith

**Affiliations:** 1 Jiangsu Key Laboratory of Biodiversity and Biotechnology, School of Life Sciences, Nanjing Normal University, Nanjing, Jiangsu Province, China; 2 Montgomery Botanical Center, Miami, FL, United States of America

**Keywords:** *Taxus wallichiana* var. *mairei*, Natural regeneration, Seed dispersal, Ex-situ conservation, Botanical garden management

## Abstract

Chinese yew (*Taxus wallichiana* var. *mairei*) is ranked as a rare and endangered plant of first-grade protection of China. It has been widely cultivated in 17 provinces of China over the past few decades. However, little is known about the dispersion, rewilding, and ecological influence of Chinese yew’s offspring during cultivation. Here, we report a noteworthy case of this species, *via ex situ* conservation, which has successfully spread into different secondary forests, thus forming a stable regenerating population in eastern China. The establishment of this yew population, which has > 900 individuals and 7 ha area, can be ascribed to two key ecological factors: (1) secondary forest near the parent yews that provided suitable microhabitats in which progeny yews could germinate and grow, and (2) seed-foraging and transportation by native birds. Thus, this case may offer a pathway for conserving endangered Chinese *Taxus* species, which can attract frugivorous birds to disperse their seeds. In addition, it is necessary to monitor the growth performance of progeny population in the field.

## Introduction

*Taxus wallichiana* var. *mairei*, known as the Chinese yew, is an evergreen dioecious tree belonging to the Taxaceae family (Fu, Li & Mill, 1999). The slow-growing tree, with a hard, tightly textured stem, can provide high-quality wood. Due to its beautiful tree form, it is often planted in the courtyard as a valuable ornamental tree. Furthermore, Chinese yew is regarded as an important woody medicinal plant because it contains taxol as a natural anti-tumor drug with unique physiological functions in bark, branches and leaves (Sun et al., 2015). The mature Chinese yews form arils which cover the seeds. The aril, similar to the fleshy fruit of angiosperms, is bright red in color and has high soluble sugar with a sweet taste (Li et al., 2017a). The aril can attract birds to feed, thus helping to transport and spread seeds (Li et al., 2017b).

**Figure 1:**
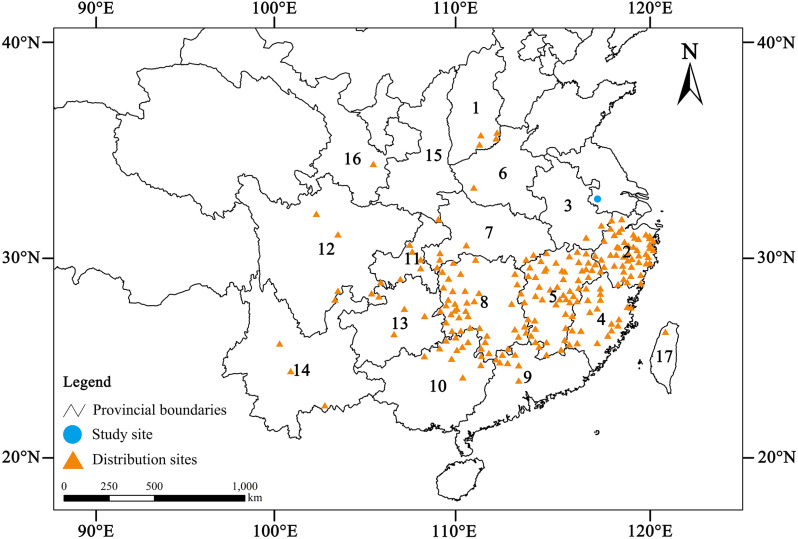
Geographic distribution of wild Chinese yew (*Taxus wallichiana* var. *mairei*) (Data from Fu, Li & Mill, 1999; Ma & Zhang, 2009; Yin, 2013; Wang & Zhang, 2019). 1. Shanxi, 2. Zhejiang, 3. Anhui, 4. Fujian, 5. Jiangxi, 6. Henan, 7. Hubei, 8. Hunan, 9. Guangdong, 10. Guangxi, 11. Chongqing, 12. Sichuan, 13. Guizhou, 14. Yunnan, 15. Shaanxi, 16. Gansu, 17. Taiwan.

Chinese yews are usually scattered or present patchy distribution on both sides of the mountain valleys, on the hillside and beside villages, 300–1,800 m above sea level (Ma & Zhang, 2009). Most of them occurred in the subtropical region of China: from Taiwan in the east to Yunnan in the southwest, and from Henan and Shanxi in the north to Guangxi and Guangdong in the south (Fig. 1). However, Chinese yew has low seed yield, low seed germination rate and survival rate of seedlings, relatively slow growth, and low genetic diversity, so it is difficult to form a large range of natural communities (Ru et al., 2006; Liu et al., 2019). The yews can be associated tree species in the deciduous forests, evergreen forests, broad-leaved forests, or even bamboo forests. This species grows well in warm and humid climates with well-drained, weakly acidic soils (pH ≈ 6.0) (Ru et al., 2006; Song, 2013; Li et al., 2014a). In the past several decades, excessive logging and agricultural reclamation have not only sharply reduced its population size, but also severely damaged its habitats (Yin, 2013). For this reason, this species was decreed a first-class national protected plant and listed on the checklist of *The Important Wild Plants for Conservation in China (first passel*) in 1999 (Yu, 1999). In addition, it is also considered to be “Vulnerable” (VU) according to the most recent Chinese Red List (Qin et al., 2017).

Currently, there are two protection strategies of Chinese yews in China. They are *in situ* conservation and *ex situ* conservation (Primack, Ma & Jiang, 2014). The former is to establish nature reserves at the provincial or national level, which is usually a protected area of forest ecosystem or wild plants. For example, The Provincial Nature Reserve of *Taxus wallichiana* var. *mairei* and *Keteleeria pubescens* in Guiyang, Hunan Province was established in 2013, and it harboured as many as 10,000 Chinese yews within the area of 744 ha (Tang, 2014). The latter is to provide care for the yews in botanical gardens, arboretums, and germplasm gardens. For example, there are a large number of young yews vigourously growing in a Chinese yew planting base in Gangxia Town, Wuxi City, Jiangsu Province, China (Gao, 2006).

Chinese yew has been widely cultivated in 17 provinces since the 1950s in China (Gao, 2006; Zhang, Li & Hou, 2018). The current studies regarding its cultivation have mainly focused on the seed collecting and germination, seedling-raising, containerized seedling, the cuttage seedling and other cultivation techniques on how to improve the taxol content during the culture (Ru et al., 2006; Xu, 2015). For example, Yu et al. (2012) reported that it would be beneficial for Chinese yew to accumulate three active components (*i.e.,* taxol) through appropriately increasing sunshine during its artificial cultivation. In contrast, little attention has been paid to the relationship between Chinese yew and local animals. Previous studies are mainly about the animal species feeding on its seeds with arils, the transporting and storing behavior of frugivore birds, and the effect of seed-consuming by such fruit-eating birds on the recruitment of seedlings of Chinese yew (Lu, Zhu & Deng, 2008; Deng, Chen & Lu, 2010; Li et al., 2020). However, little is known about the dispersion, rewilding, and ecological influence of Chinese yew’s offspring during cultivation.

Here, we reported a noteworthy case of Chinese yews which were cultivated in a botanical garden in eastern China. First, we made a field demographic survey on parent trees of Chinese yew and their progenies nearby. We then compared the progeny yews’ abundance, population structure, distribution area, and regeneration strategy in two different forest types not far away from the parent yews. Finally, we critically evaluated what caused the rewilding of the population, discussed the implications for the yews’ conservation and expounded the influence of expanding population of progeny yews on local diversity.

## Materials & Methods

### Study area

The research area was located in the Nanjing Botanical Garden Memorial Sun Yat-Sen (hereafter, NBG; 31°14′∼32°37′N, 118°22′∼119°14′E; 30∼60 m a.s.l) and the parts of neighboring Purple Mountain, eastern China (Liao et al., 2014). The botanical garden covers 186 ha, including 100 ha natural vegetation reserves which are connected with the forest vegetation of the Purple Mountain. The area belongs to the north subtropical monsoon climate. The annual average precipitation is 1000.4 mm, the annual average temperature is 14.7 °C, and the annual frost-free period is 237 days (Li et al., 2014a). The main topography is a hillside, and the soil type is dominated by yellow-brown soil within the forest.

According to our field survey, the parent trees of Chinese yew grow at the Pinetum of NBG, and all of them are on flat ground. Li, He & Sheng (1999) noticed a regenerating population with 461 Chinese yews which occurred in *Quercus acutissima* or *Pterocarya stenoptera* community at NBG, eastern China. Liao et al. (2014) reported that the yew population increased to more than 700 individuals in the secondary forests which were located at NBG and on mountainous slope of the Purple Mountain. Zhang, Li & Hou (2018) found that most of the progeny yews (> 800 individuals) grew better under a single neighboring tree (height ≥ 10 m) than under two, three or zero neighboring trees.

**Figure 2:**
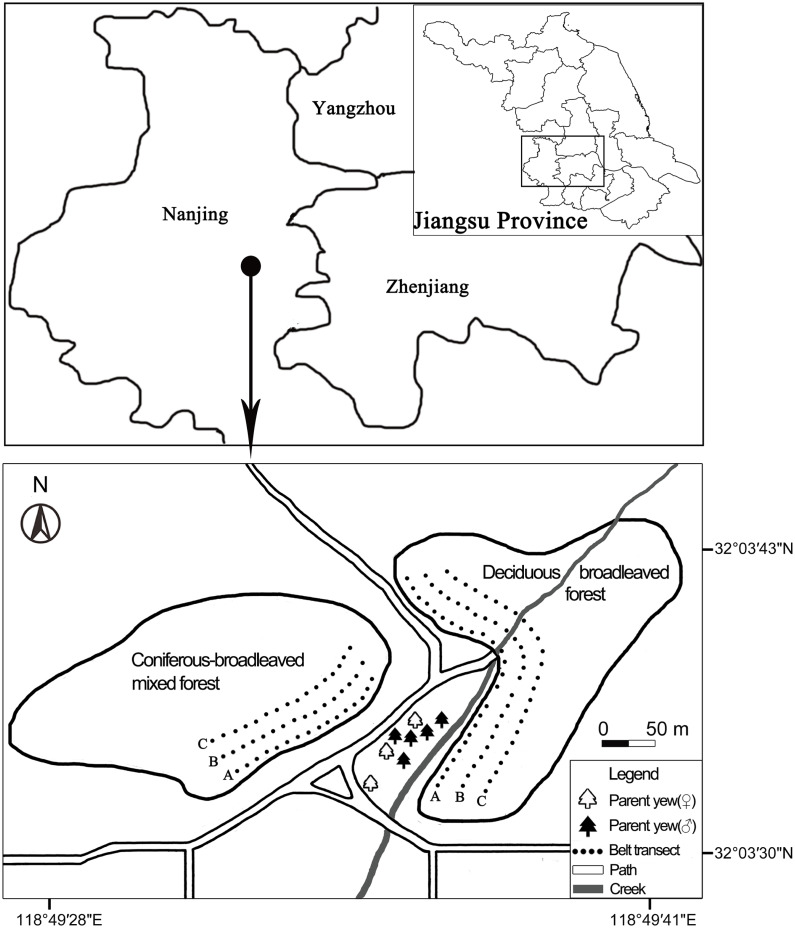
Distribution areas and sampling plots of naturally regenerating yew (*Taxus wallichiana* var.* mairei*) populations at the Nanjing Botanical Garden and Purple Mountain, eastern China. Each dot represents one sampling plot (20 m × 20 m) of three contiguous belt transects, which were arranged from near to far distances from the parent yews in deciduous broadleaved forest (*n* = 25 plots) and coniferous-broadleaved mixed forest (*n* = 16 plots), respectively.

These progeny yews have successfully disseminated into different secondary forests of the nearby hillside, thus forming a regenerating population therein (Zhang, Li & Hou, 2018). There is a creek east of the parent yews, near which there are divergent paths (Fig. 2). These forests are not far away from the parent yews. They can be divided into two forest types: deciduous broadleaved forest (DB) and coniferous-broadleaved mixed forest (CB) at NBG. The dominant tree species in DB are *Quercus variabilis*, *Pterocarya stenoptera*, *Liquidambar formosana*, and *Cryptomeria japonica* var. *sinensis* while in CB are *Celtis sinensis*, *Cinnamomum camphora*, and *Cephalotaxus fortunei*.

In addition, due to diverse habitat and various vegetation, more than 70 species of forest birds are found in this area (Zhang et al., 2018).

### Field sampling

Field work was approved verbally by Mr. Yuning Xiong at NBG. In June of 2020, we conducted a field survey on Chinese yew cultivated (parent yews) in the botanical garden and their regenerating populations (progeny yews). No wild Chinese yew populations exist in Jiangsu Province (Liu, 2015). However, in the 1950s, eleven seedlings of Chinese yews were introduced to the Pinetum at NBG from Lushan Botanical Garden in Jiangxi Province. Eight adult Chinese yews survived and were planted in the garden intensively (Li et al., 2014a). The sex of each parent trees was recorded, and their DBH (diameter at 1.3 m above the ground), tree height and first branch height were measured using a caliper, tape meter, or contracting-height-meter as needed and following standard field procedures (Li & Zhang, 2015).

The deciduous broadleaved forest on a mountain slope is located in the east of the parent yews. A small creek runs through the DB forest. In the west of parent yews is the coniferous-broadleaved mixed forest, which is separated from its parent trees by a path (Fig. 2). We recorded each progeny yew’s number, DBH, height, first branch height, and regenerating mode (true seedling *vs* sprouting) in both forests by applying two approaches. First, we set three contiguous belt transects in each forest type. The transects were marked as A, B, and C; they were arranged from near to far by the distance from the parent yews in deciduous broadleaved forest (25 plots) and coniferous-broadleaved mixed forest (16 plots) respectively (Fig. 2). The plot size was 20 m × 20 m in all transects. Second, we found and recorded the same items for every yew outside the plots but within each forest type. Finally, we also measured the distribution areas of progeny yews in each forest type by using a GPS Tool Box (https://sourceforge.net/projects/gpstoolbox/).

**Figure 3:**
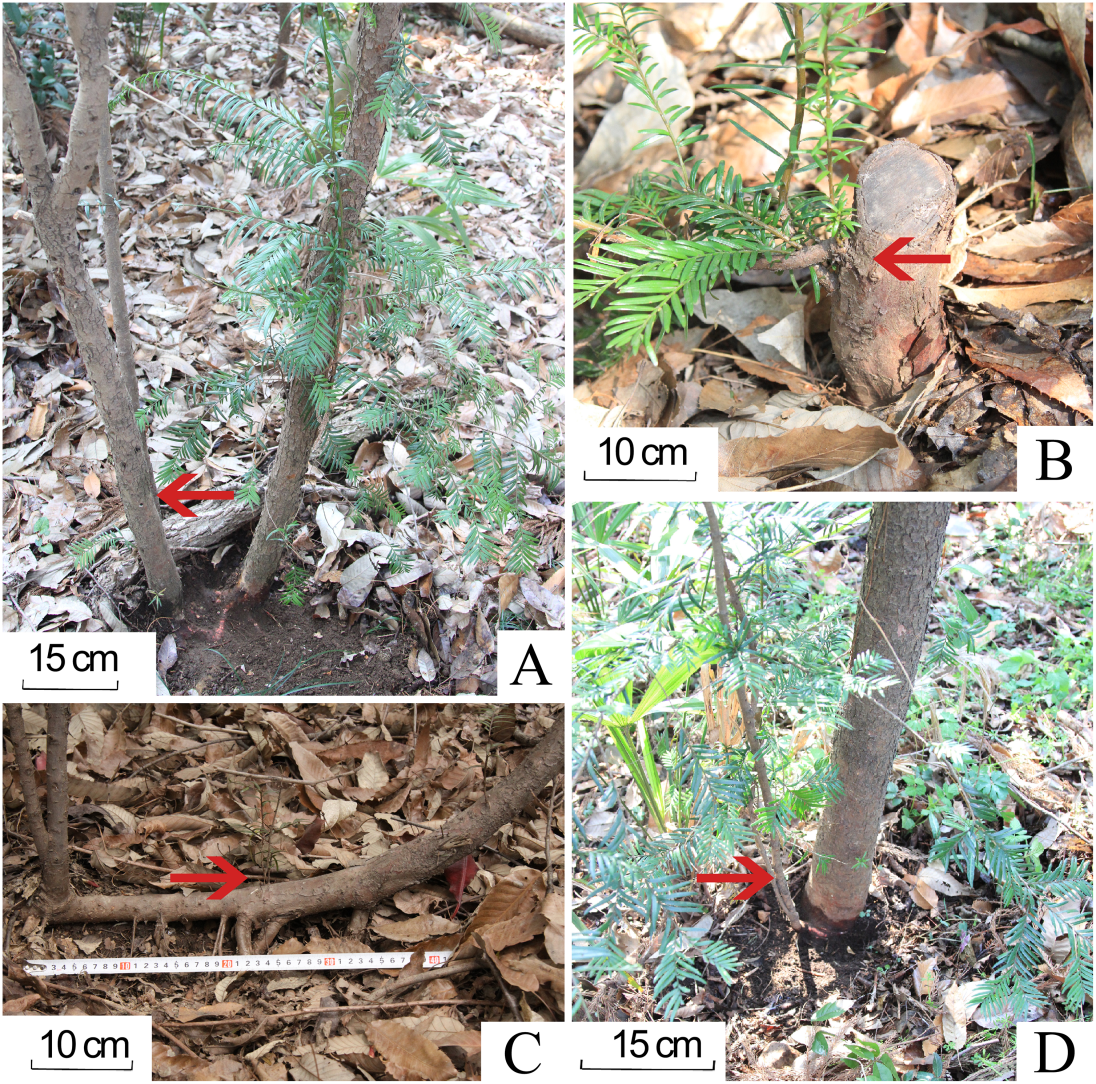
Four types of sprouting in progeny Chinese yews in the studied forest stands. (A) sprouting from roots. (B) crown sprouts. (C) opportunistic sprouts. (D) collar sprouts. The photographs were taken by Guangfu Zhang.

In addition, the plantlets of progeny Chinese yew were classified into two categories: true seedlings from seed-origin and sprouts from adventitious/dormant buds on lateral roots, root collar, stubs, or drooping branches, identified by removing the litter and excavating underground root system (Fig. 3). Individual yew with own root system in the plot was also considered to be seed-orientated (Ky-Dembele et al., 2007; Shang et al., 2020).

### Data processing

Individual growth status of Chinese yew was described by DBH, height, and first branch height. Live crown ratio (LCR) was used to report the shade tolerance of Chinese yews. Furthermore, LCR was calculated as follows: LCR = ([tree height - first branch height]/tree height). For convenience, we used the height of a tree minus the first branch height to represent tree crown vertical length (Zhang, Li & Hou, 2018).

Diameter structure of progeny yew population was divided according to the actual measured DBH and combined with its life history characteristics (Li et al., 2014a; Liang & Wei, 2020):

I, DBH < 2.5 cm (seedlings);

II, 2.5 cm ≤ DBH < 5 cm (saplings);

III, 5 cm ≤ DBH < 7.5 cm;

IV, 7.5 cm ≤ DBH < 10 cm;

V, 10 cm ≤ DBH;

Likewise, these progeny yews were also divided into five categories in terms of tree height (Huang, Li & Su, 2000; Ahmadi et al., 2020):

I, H < 2 m;

II, 2 m ≤ H < 4 m;

III, 4 m ≤ H < 6 m;

IV, 6 m ≤ H < 8 m;

V, 8 m ≤ H.

We applied the two-sample *t* test to compare the differences in mean DBH, tree height, and first branch height of the Chinese yews between the CB forest and DB forest. We also used one-way analysis of variance (ANOVA) to compare the number, DBH, and height growth of Chinese yew among the three different belt transects in each forest type (Xue, 2011). Basic data processing and analysis were performed in MS-Excel 2016 and SPSS v22 software (SPSS Inc., Chicago, USA).

## Results

According to our field survey, there were three females and five males of Chinese yew in the Pinetum. As parent trees, they all grew vigorously. The average live crown ratio of parent trees was 0.78 ± 0.03. For the average DBH, the females were larger than the males. In terms of the average height and first branch height, the females were shorter than the males (Table 1). On the basis of historical record, the parent trees were about 65 years old, and they began to produce seeds in the 1990s. Based on field observations of recent years (G. Zhang, 2020, pers. comm.), the female yews produced considerable seeds every year, depending on the mast year or not.

**Figure 4:**
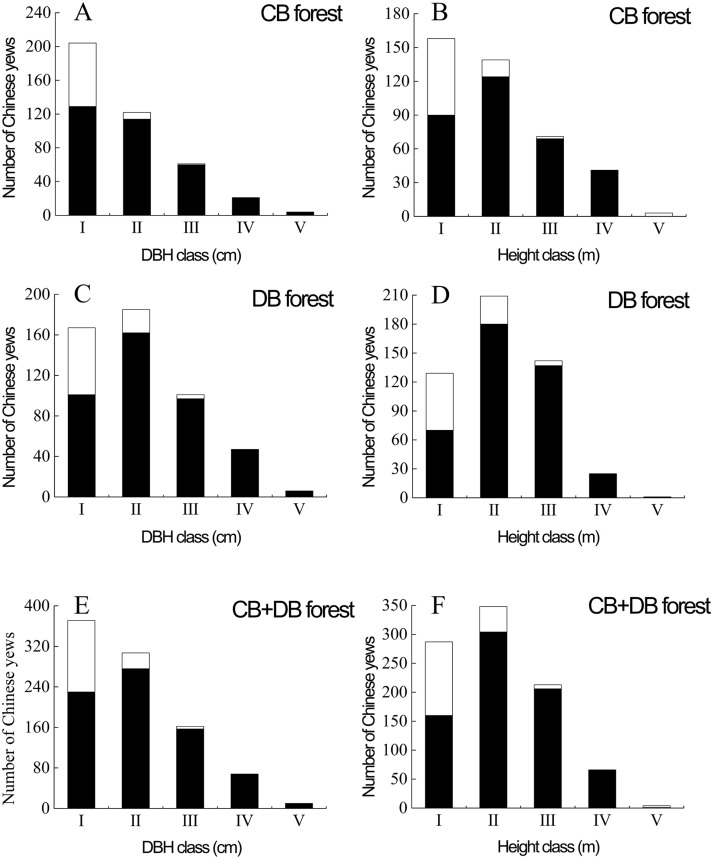
Population structures of sprouting (white) and true seedling (black) Chinese yews in DB and CB forests at the Nanjing Botanical Garden and Purple Mountain, China, respectively. The Roman numerals (I–V) stand for population structure of progeny yews in DBH class (in A, C, E) and height class (in B, D, F) respectively. I, DBH < 2.5 cm; II, 2.5 cm ≤ DBH < 5 cm; III, 5 cm ≤ DBH <7.5 cm; IV, 7.5 cm ≤ DBH <10 cm; V, 10 cm ≤ DBH. I, *H* < 2 m; II, 2 m ≤ *H* < 4 m; III, 4 m ≤*H* < 6 m; IV, 6 m ≤*H* < 8 m; V, 8 m ≤ H. DB, deciduous broadleaved forest; CB, coniferous-broadleaved mixed forest.

There were 506 and 412 progeny yews in DB and CB forest respectively. These yews had developed into a naturally regenerating population. The progeny yews in both stands regenerated in two ways: as true seedlings (80.72%) and *via* sprouting (19.28%) (Fig. 4). In the CB forest, yews had 84 sprouts, taking 20.39% of the total number of 412 yews; in the DB forest, yews had 93 sprouts, taking 18.38% of the total number of 506 yews. Most sprouting yews occurred in the stage of seedlings (DBH < 2.5 cm) in both stands. In each forest type, regeneration *via* sprouting included four types: sprouts from the roots, collar sprouts from the base of the trunk, crown sprouts from the stub or stem aboveground, and opportunistic sprouts from layered branches (Fig. 3).

As shown in Table 2, the distribution area of regenerating population in DB forest was 20365 m^2^ while that was 58170 m^2^ in CB forest. The number of progeny yews in DB forest was 506, more than that in CB forest (412). Furthermore, the value of mean DBH, height, and first branch height of progeny yews in DB forest were greater than those in CB forest except LCR. For example, the mean DBH in DB forests (*n* = 506) and in CB forest (*n* = 412) were 3.89 ± 0.12 cm and 3.08 ± 0.12 cm respectively, and the difference was statistically significant (two-sample *t* test: *t* = 4.812, *df* = 916, *P* = 0.00) (Table 3), indicating that the yews in DB forest grew better than in CB forest. For these yews of DBH ≥ 5 cm, their mean height and LCR in CB were greater than those in DB forest respectively (two-sample *t* test: *t* =  − 5.023, *df* = 238, *P* = 0.00; *t* =  − 2.901, *df* = 238, *P* = 0.004); there was no significant difference in their mean DBH or first branch height between in CB and DB forest (two-sample *t* test: *t* = 1.041, *df* = 238, *P* = 0.299; *t* = 0.341, *df* = 238, *P* = 0.734).

**Table 1:** Growth status of eight parent Chinese yews at the Nanjing Botanical Garden, eastern China.

**Parent Chinese yews**	**Number**	**Average DBH (cm)**	**Average tree height (m)**	**Average first branch height (m)**	**Average live crown ratio**
Female	3	31.71 ± 4.09	10.67 ± 0.27	1.96 ± 0.34	0.81 ± 0.04
Male	5	28.25 ± 2.46	10.25 ± 0.62	2.38 ± 0.19	0.76 ± 0.03
Total	8	29.55 ± 2.08	10.41 ± 0.40	2.22 ± 0.18	0.78 ± 0.03

**Notes.**

Numbers after “±” indicate standard deviations.

Their growth conditions were very similar in terms of geographical position, altitude, slope, and the farthest distance from parent trees as well (Table 2). The number of progeny yews in both forests decreased gradually from transect A to B till C (ANOVA: *F*_2,120_ = 20.035 > 3.072, *P* = 0.000 < 0.001; Table 4), indicating that the further from parent trees, the fewer progeny yews.

The population structure of progeny Chinese yew was expanding population based on its size-class distribution in all sample areas (Fig. 4). In terms of the DBH, most individuals belonged to the first and second size classes in the two forest types. In addition, the yews with DBH < 5.0 cm amounted to 326 individuals (79.13%) in the DB forest, and 352 individuals (69.57%) likewise in the CB forest. In other words, these yews were predominated by seedlings and saplings in both forests. For height, most individuals fell into the first and second classes in CB forest. However, in DB and total forest most individuals were included in the first three classes, indicating the majority of yews less than six m in height (Fig. 4). In terms of DBH and height respectively, the adult yews in the two stands only had 240 individuals (26.14%)(DBH ≥ 5 cm), 68 ones (7.41%) (H ≥ 6 m). Collectively, the size-class distribution of tree height was similar to that of DBH.

## Discussion

### What factors contribute to spreading and rewilding of progeny yew populations?

In this study, we report a noteworthy case of endangered species, *i.e.,* the Chinese yews (*Taxus wallichiana* var. *mairei*). The seeds of parent yews at NBG, eastern China can be dispersed to the secondary forests nearby, then they germinate and grow therein, thus unexpectedly developing a rewilding population (Table 2).

**Table 2:** Characteristics of two forest types with naturally regenerating Chinese yew populations at the Nanjing Botanical Garden and Purple Mountain, eastern China.

**Item**	**Deciduous broadleaved forest**	**Coniferous-broadleaved mixed forest**
East longitude	118°49′36.10″∼47.64″	118°49′24.85″∼36.79″
North latitude	32° 03′29.50″∼44.86″	32° 03′30.96″∼40.14″
Altitude (m)	41∼74	47∼63
Slope angle (°)	30	35
Direction of slope	SW	SE
Yew distribution area (m^2^)	20365	58170
Number of yews	506	412
Largest yew in DBH (cm)	17.90	12.29
Highest yew (m)	8.70	8.50
Farthest distance from parent yews (m)	350	365

**Table 3:** The mean DBH, height, first branch height, and live crown ratio of two forest types of *Taxus wallichiana***var.***mairei* in the Nanjing Botanical Garden and Purple Mountain, eastern China.

**Forest type**	**Mean DBH (cm)**		**Mean Height (m)**		**Mean first branch height (m)**		**Mean live** **crown ratio**	
	DBH ≥ 5 cm	Total	DBH ≥ 5 cm	Total	DBH ≥ 5 cm	Total	DBH ≥ 5 cm	Total
CB forest	6.92 ± 0.16^a^ (*n* = 86)	3.08 ± 0.12^b^ (*n* = 412)	5.69 ± 0.13^a^ (*n* = 86)	2.92 ± 0.09^b^ (*n* = 412)	1.20 ± 0.10^a^ (*n* = 86)	1.20 ± 0.04^b^ (*n* = 412)	0.65 ± 0.02^a^ (*n* = 86)	0.58 ± 0.01^a^ (*n* = 412)
DB forest	7.18 ± 0.16^a^ (*n* = 154)	3.89 ± 0.12^a^ (*n* = 506)	4.96 ± 0.08^b^ (*n* = 154)	3.19 ± 0.07^a^ (*n* = 506)	2.04 ± 0.0 7^a^ (*n* = 154)	1.47 ± 0.08^a^ (*n* = 506)	0.58 ± 0.01^b^ (*n* = 154)	0.5 ± 0.05^a^ (*n* = 506)

**Notes.**

DB forest, deciduous broadleaved forest; CB forest, coniferous-broadleaved mixed forest; n, number of Chinese yew in each forest type. Numbers after “±” indicate standard errors; different letters indicate significant differences (*P* < 0.05) within columns.

**Table 4:** The average yews density (no./400 m^2^) in two forest types of *Taxus wallichiana* var. *mairei* in three transects at the Nanjing Botanical Garden and Purple Mountain, eastern China.

**Transect**	**Yews in DB forest**			**Yews in CB forest**			**Yews in DB+CB forest**		
	DBH < 5 cm	DBH ≥ 5 cm	Total	DBH < 5 cm	DBH ≥ 5 cm	Total	DBH < 5 cm	DBH ≥ 5 cm	Total
A	8.72 ± 1.80^a^	4.64 ± 1.03^a^	13.68 ± 2.67^a^ (*n* = 342)	7.38 ± 2.36^a^	3.63 ± 1.19^a^	11.00 ± 2.91^a^ (*n* = 176)	8.20 ± 1.42^a^	4.24 ± 0.77^a^	12.63 ± 1.97^a^ (*n* = 518)
B	3.56 ± 0.92^b^	0.92 ± 0.24^b^	4.48 ± 1.05^b^ (*n* = 112)	6.00 ± 0.79^ab^	1.44 ± 0.49^b^	7.44 ± 1.16^ab^ (*n* = 119)	4.51 ± 0.66^b^	1.12 ± 0.24^b^	5.63 ± 0.81^b^ (*n* = 231)
C	0.76 ± 0.31^b^	0.40 ± 0.20^b^	0.96 ± 0.42^b^ (*n* = 24)	2.00 ± 0.53^b^	0.50 ± 0.27^b^	2.50 ± 0.75^b^ (*n* = 40)	1.24 ± 0.29^c^	0.44 ± 0.16^b^	1.56 ± 0.40^c^ (*n* = 64)

**Notes.**

DB, deciduous broadleaved forest; CB, coniferous-broadleaved mixed forest; the transect in DB forest has 25 plots while in CB has 16 ones; n, number of yews. Numbers after “±” indicate standard errors; different letters indicate significant differences (*P* < 0.05) within columns.

The Chinese yew usually grows in damp valleys, hillsides, and open slopes in China (Fu, Li & Mill, 1999; Ru et al., 2006). As a shade-tolerant tree species, it occurs in the wild in coniferous forests, evergreen broad-leaved forests, and conifer-broadleaved mixed forests. Most dominant tree species in these forests—such as *Liquidambar formosana*, *Fagus longipetiolata*, *Castanopsis sclerophylla*, *Quercus glauca*—may serve as nurse plants, being able to provide suitable environment for yews to survive and grow in (Ma & Zhang, 2009; Zhang, Li & Hou, 2018). In the current study, both sampled forest stands (CB and DB) are now more than 70 years old. Most of these tree species thus have a DBH > 40 cm. These forests are located on low mountain slope, with good drainage and little human interference, and their soils are deep and contain rich organic matter (Li et al., 2014a). Accordingly, all these features should create favorable conditions for progeny yews to establish and grow. This is the first leading factor for regenerating population arising from parent yews nearby.

The eight parent yews at the Pinetum of NBG have DBH > 30 cm, on average, with tree heights of > 10 m (Table 1), and their tree age is just 65 years old. By contrast, wild yews can attain a DBH > 100 cm, with the corresponding ages between 1000 and 1600 years old (Nan et al., 2009). Because of their slow woody growth, Chinese yews need to take more than 35 years to start bearing seeds themselves (Ru et al., 2006). At present, the three yews grow vigorously, and they presumably have produced seeds yearly since the 1990s (Zhang, Li & Hou, 2018). As a result, in theory they are able to produce considerable seeds yearly. In addition, the Pinetum is not too far away from the sampled forests (at < 400 m) (Table 2). Therefore, the vigorously reproductive yews should be able to offer abundant seeds every year for dispersal.

Animal-mediated seed dispersal is the second key factor to consider. Birds consume and spread the seeds of the yews at NBG, which have a fleshy aril, are sticky, sweet, and nutritious (Liu et al., 2012). The yews’ arils turn red in ripe seeds. Generally, red is the most striking color that foraging birds notice (Gagetti, Piratelli & Piña Rodrigues, 2016). Lu, Zhu & Deng (2008) found that six bird species (*Urocissa erythrorhyncha*, *Cyanopica cyana*, *Pycnonotus sinensis*, *Zoothera dauma*, *Turdus naumanni* and *Turdus hortulorum*) ingesting their seeds at Chinese yews’ tree, and demonstrated that frugivorous birds play a significant role in establishing of regenerating population of this yew in *ex situ* conservation. There is a wide range of secondary forests and various birds found at the Purple Mountain, with previous studies showing that *U. erythrorhyncha* and *P. sinensis* are the major vectors to forage and spread ripe seeds of Chinese yews at the NBG (Lu, Zhu & Deng, 2008; Zhang, Li & Hou, 2018). Moreover, compared with *Pycnonotus sinensis*, *Urocissa erythrorhyncha* is more active. When they forage for seeds, they may fly to forests 300–400 m away from the parent yews (Deng, Chen & Lu, 2010). In addition, a creek bisects the sampled DB forest (Fig. 2), which also may influence bird movements. The two forest birds often fly, forage, perch in a small flock (especially having three to five), and they like to live near water sources such as a creek, or river. Hence, we posit that this plant-animal interaction may result in more progeny yews in DB forest than in CB forest.

More recently, Li et al. (2020) considered that rodents played a significant role in seed removal of Chinese yews in Tianmu Mountain, Zhejiang Province. Moreover, the scatter-hoarding and re-caching by rodents can promote long-distance seed dispersal (Wang et al., 2019). It is reported that the rodents in the Purple Mountain can forage the nuts of three species of *Quercus* (Liu et al., 2016). Thereby, we speculate that the rodents in this area may also feed on the seeds of Chinese yew, thus possibly contributing to seed dispersal and seedling establishment.

Therefore, the establishment of the yew population, with > 900 individuals among 7 ha (Table 2), can be ascribed to two key factors. Yew seeds are foraged and transported by birds, and secondary forest near the parent yews provides suitable habitat for the progeny yews to germinate and grow in.

### Implication for *ex situ* conservation and mangement

In the past three decades, Chinese yews *via ex situ* conservation have spread into different secondary forests at the NBG and nearby Purple Mountain, developing an expanding population and increasing in size and distribution (Zhang, Li & Hou, 2018). Although currently these yews have not yet born seeds themselves according to our field survey, we predict the regenerating population to blossom and yield seeds in 2025. Therefore, the progeny yews could be considered as a self-sustaining and rewilding population in the near future.

All of the seven wild species of *Taxus* including Chinese yew have been decreed first-class national protected plants since 1999 (Yu, 1999). However, there is no wild Chinese yew in Jiangsu Province (including Purple Mountain) (Fig. 1). In this study, we documented its population size, distribution area, regeneration mode and population structure of the rewilding population in detail. These results not only can provide important basic data for future protection and management of Chinese yew, but also provide a pathway for conserving other endangered species from *Taxus* in China. By virtue of the interaction between an introduced plant and local animals in the locality of *ex situ* conservation, its propagules (such as seeds or fruits) may be dispersed into nearby suitable habitats to form a new regenerating population, which can be a beneficial supplement to *ex situ* conservation.

At the same time, despite the lack of actual evidence of impact on local diversity, we think that the expansion of this rewilding population is likely to affect the species diversity of secondary forests in the Purple Mountain, Nanjing.

### (1) To influence regeneration of understory seedlings in secondary forests

As a shade-tolerant tree species, the seedlings of progeny yew will benefit from the deep and fertile soil under secondary forest, which is conducive to their growth (Xu, 2015). At present, the seedling and sapling yews in DB and CB forest is both more than 300 individuals (Table 3). This will have a serious effect on species, abundance, regeneration mode and growth performance of understory seedlings.

### (2) To influence progressive succession of secondary forests

Chinese yew is an evergreen tree species. Now the biggest yew is 8.7 m high and 17.9 cm in DBH in the plots (Table 2). On the one hand, they may require more environmental resources (*i.e.,* light, water and space, etc.) when they continue to grow, thus making strong intraspecific competition among these yews. On the other hand, these yews may suffer from intensive interspecific competition from their associated tree species with the increase in tree diameter. Inevitably, these competitions will further affect the progressive succession of current secondary forests.

### (3) To influence local fruit-consuming birds and native plants

At present, there are more than 70 species of birds in the Purple Mountain, and six of them are found to feed on Chinese yews’ seeds (Lu, Zhu & Deng, 2008; Zhang, Li & Hou, 2018). Of them, *Urocissa erythrorhyncha* and *Pycnonotus sinensis*, are the two most common forest birds which are able to swallow yew seeds with arils (Li et al., 2014a; Li et al., 2014b). Actually, besides yews they also forage and consume the fruits or seeds of other species like *Cinnamomum camphora*, *Morus alba*, *Broussonetia papyrifera*, and so on (Li & Yin, 2004; Lu & Lu, 2019). If the progeny yews begin to bear seeds, whose aril are sweet, red and nutritious, thus making them attract these birds. Not surprisingly, the seeds (with arils) of Chinese yews can attract more birds than local or native plant species, which may probably cause apparent competition. If so, it will affect the propagation, distribution and growth of native plants in the long run.

To date, no management actions have been performed regarding the offspring populations of Chinese yews in the region. Therefore, based on our document in this case, we highly recommend that (1) attention should be paid to the growth and spread of this rewilding population; (2) such population should be included in the Botanical Garden’s management objectives of cultivated plants, and should simultaneously be incorporated in protection system of secondary forests in Purple Mountain as soon as possible.

## Conclusions

In summary, we report a noteworthy case in which the progeny of Chinese yew (*Taxus wallichiana* var. *mairei*) *via ex situ* conservation from a botanical garden in eastern China formed a rewilding population in two different secondary forests nearby. This was mainly due to continuous provenance and suitable habitats. Furthermore, our study highlights the need to consider the interaction between native herbivores and cultivated plants when introducing exotic plants in botanical gardens in the future. The conservation practice in this case may also be applied to other endangered *Taxus* species in botanical gardens, arboretums, and the like, because they can attract frugivorous birds to disperse their seeds. At the same time, it is necessary to monitor the population growth of progeny yews in the coming future, and simultaneously to concern about the influence of their population dispersion on local diversity.

##  Supplemental Information

10.7717/peerj.12341/supp-1 Supplemental Information 1 Raw data of Chinese yews in NBG Raw data of parent yews, yews in CB forest, and yews in DB forest respectively. These data were used for statistical analysis to compare CB forest and DB forest.

## Additional Information and Declarations

### Field Study Permissions

The following information was supplied relating to field study approvals (i.e., approving body and any reference numbers): Field experiments were approved by the Nanjing Botanical Garden Memorial Sun Yat-Sen (hereafter, NBG). No written permission is required for field work in this area. Therefore, we got verbal permission which came from Mr. Yuning Xiong at NBG.

### Data Availability

The following information was supplied regarding data availability: The raw measurements are available in the Supplementary File.
